# Enhancing the Usability of the Health Declaration System Upon Entry (Q-CODE) through Private Platform Integration

**DOI:** 10.56786/PHWR.2026.19.43.3

**Published:** 2026-11-12

**Authors:** Seongjoo Yu, Daeil Park, Min-Kyung Chae, Hyojun Ju, Kanghyeon Lee, Seongsun Kim

**Affiliations:** 1 Division of Epidemiological Data Analysis, Department of Data Science, Korea Disease Control and Prevention Agency Cheongju Korea; 2 Division of Quarantine Policy, Department of Infectious Disease Emergency Preparedness and Response, Korea Disease Control and Prevention Agency Cheongju Korea

**Keywords:** Quarantine, Communicable diseases, Public-private sector partnerships, Personal Health Records

## Abstract

**Objectives:**

The Korea Disease Control and Prevention Agency (KDCA) has operated the Health Declaration System Upon Entry (Q-CODE) since 2022, which allows travelers required to declare quarantine requirements under the “Quarantine Act” to electronically submit required information before entry, including travel history, places of stay and transit, and health status. To improve accessibility and usability, the KDCA began integrating Q-CODE with private platforms in 2025. This study aimed to describe the integration process, expanded access channels, initial use, and future challenges.

**Methods:**

On December 15, 2025, Q-CODE launched an integration with Naver that linked personal information fields to users’ Naver account data. In June 2026, we added access through the Personal Health Records (PHR) application. We analyzed 148,861 quarantine declarations submitted from June 1 to July 31, 2026, by submission method.

**Results:**

The Naver integration reduced the required manual entries from 11 to 5. Of 148,861 declarations, 6,553 (4.4%) were submitted via Naver: 3,053 (4.1%) in June and 3,500 (4.8%) in July.

**Conclusions:**

Integrating with Naver, a private platform, reduced direct user input and expanded Q-CODE’s online access channels from the KDCA website to Naver and the PHR application. The existing Q-CODE website remained accessible without separate login or authentication to avoid user confusion. Integration with more private platforms is warranted, while preserving optional, universal access for all users regardless of platform.

**Key messages**

**① What is known previously?**

The Korea Disease Control and Prevention Agency operates Health Declaration System Upon Entry (Q-CODE), which allows travelers to submit health information electronically before entering the Republic of Korea for infectious disease screening.

**② What new information is presented?**

Integrating Naver with Q-CODE cut manual entries from 11 to 5. In June and July 2026, 6,553 (4.4%) of 148,861 quarantine declarations were submitted via Naver.

**③ What are implications?**

Integrating widely used private platforms with public quarantine services can expand access and improve convenience.

## Introduction

With the increase in overseas travel and the normalization of cross-border movement, quarantine has become increasingly important for the early identification of and response to infectious diseases imported from overseas. The Korea Disease Control and Prevention Agency (KDCA) collects information from entrants required to submit declarations under the “Quarantine Act”, including their areas of stay and transit and health status. This information is used to implement necessary public health measures, including quarantine investigations at the point of entry and subsequent contact tracing [1].

During the early stages of the coronavirus disease 2019 (COVID-19) pandemic, measures to prevent the initial introduction of COVID-19 into the Republic of Korea (ROK) and subsequent community transmission included temperature screening, submission of health questionnaires and special quarantine declaration forms, and post-entry monitoring for all entrants. As the pandemic spread domestically and internationally, additional measures were introduced, including requirements for pre-entry negative test certificates and quarantine at home or in temporary residential facilities, depending on vaccination status. Subsequently, all countries worldwide were designated as quarantine management areas, necessitating the collection of paper-based health questionnaires from all entrants. Consequently, the number of individuals subject to quarantine procedures increased substantially. Collecting paper documents and conducting quarantine investigations for all entrants required considerable time, inconvenienced entrants, and increased the workload of quarantine officers.

Accordingly, the KDCA discontinued the special entry procedure using the self-quarantine app on February 21, 2022. To strengthen efforts to prevent the importation of infectious disease cases from overseas while minimizing passengers’ waiting times at entry, the KDCA introduced the Health Declaration System Upon Entry (Q-CODE) at Incheon International Airport on March 21, 2022. Q-CODE allows entrants to electronically submit information required for quarantine, including their health status, before arrival and obtain a QR code. With the introduction of kiosks in the arrivals hall and the implementation of Q-CODE, the average quarantine processing time was reduced from 2–4.5 minutes to approximately 45 seconds. Furthermore, the legal basis for submitting documents through the quarantine information system was clarified through an amendment to Article 12 of the “Quarantine Act” on January 23, 2024 [1].

In response to domestic and international developments, including the World Health Organization’s termination of the public health emergency of international concern status for COVID-19 in May 2023, the downgrading of the crisis alert level in June 2023, and the reclassification of COVID-19 as a Class 4 infectious disease, the COVID-19 crisis alert level was lowered to “Alert.” Consequently, the designation of all countries worldwide as COVID-19 quarantine management areas was lifted, along with the associated quarantine measures. According to a public perception survey conducted by the KDCA in November 2023, following the phased restoration of daily life, 76.8% of the 505 respondents who had used Q-CODE reported being satisfied with the system. This finding indicates that most respondents who had used Q-CODE reported being satisfied with the system. However, users also reported that the Q-CODE website required the completion of too many fields to obtain a QR code and had an inconvenient interface. In response, the KDCA sought to improve usability by simplifying the required fields, removing unnecessary instructions, and eliminating duplicate fields [1].

Internationally, digital services that allow entrants to submit required information before arrival, such as Japan’s Visit Japan Web and Singapore’s SG Arrival Card, are also in operation [2,3]. In light of these domestic and international developments and the ongoing digital transformation, it is necessary to consider diversifying access channels to make quarantine declaration services more accessible to users, alongside improving the functionality of Q-CODE itself. Accordingly, this report describes the integration of Q-CODE with private-sector platforms and the expansion of access channels, initiatives that have been underway since 2025. It also outlines the operational framework, analyzes initial usage patterns following the integration, and identifies areas for future improvement.

## Methods

### 1. Promoting Integration with Private-sector Platforms

To improve user convenience, the KDCA additionally explored integration with private-sector platforms. Rather than limiting the service to a single platform, the KDCA adopted an approach of engaging multiple private-sector platforms and introducing integrated services sequentially.

As the first implementation, the Naver Pre-Arrival Quaran­tine Info Service, which links the Naver Access Badge with Q-CODE, was launched on December 15, 2025 [4]. The integration was designed to improve user convenience by linking personal information fields that would otherwise require repeated entry with identity-verified Naver member information. This enabled the automatic entry of basic information, allowing users to complete the quarantine declaration by manually entering only the required quarantine-related information.

In addition, personal data protection and system stability were established as key design requirements. Q-CODE requires overseas travelers to provide personal information, such as their names and passport numbers, and sensitive information, including countries visited and health status. Before users could access the Naver Pre-Arrival Quarantine Info Service, consent was obtained for the collection and use of personal information and its provision to third parties. Separate data retention and destruction criteria were established for information stored on servers. In addition, quarantine pre-entry information stored on Naver’s servers was configured for automatic deletion at midnight on the day following the scheduled date of arrival in the ROK.

### 2. Expansion of Access Channels

Three online access channels were available: ① direct access to the Q-CODE website, ② the Naver Pre-Arrival Quarantine Info Service, and ③ the Personal Health Records (PHR) app. Added in June 2026, the PHR app channel provides access to the Q-CODE website directly from the app’s main screen. The direct website channel was retained to allow users to access the service without separate login or authentication. This approach reflects the role of private-platform integration as an optional feature intended to improve convenience for existing users, rather than making registration with a specific platform a prerequisite for submitting a quarantine declaration. Accordingly, individuals who were not members of the participating private platforms, users under 14 years of age, and foreign entrants could also submit quarantine declarations without having to register for a participating platform.

To facilitate on-site quarantine declarations in addition to online submissions, paper health questionnaires are distributed on board flights before arrival, and kiosks are installed and operated at quarantine counters in the arrivals hall. In particular, paper health questionnaires are also provided to entrants who are unfamiliar with smart devices, enabling them to complete quarantine declarations regardless of their level of digital accessibility [5].

Meanwhile, language support was tailored to the target users of each access channel. Because the Q-CODE website and arrivals hall kiosks serve as public quarantine services for entrants of various nationalities, they support multiple languages. In contrast, the Naver service was provided in Korean and English, reflecting the characteristics of its target users.

### 3. Public Outreach

To inform users about the new access channel, public outreach activities were conducted alongside the service launch. Following the launch of the Naver-linked service in December 2025, the KDCA issued a press release and collaborated with private-platform operators to produce promotional materials [4]. In July 2026, the KDCA produced and distributed card news explaining how to use the three online Q-CODE access channels and provided guidance through the PHR app (Figure 1) [6,7].

## Results

### 1. Reduction in Input Fields through Integration with Private Platforms

Following implementation, the number of fields that users must enter manually in the Naver Pre-Arrival Quarantine Info Service was reduced from 11 to 5 through linkage with Naver member information (Table 1). The six fields that no longer require manual entry are English name, passport number, address of stay, date of birth, domestic contact information, and the consent field for users under 14 years of age. Of these, English name, passport number, and address of stay did not need to be re-entered after their initial entry on the existing Q-CODE website. However, the Naver-linked service eliminates even this initial entry. For date of birth and domestic contact information, manual entry was replaced by retrieval through member information linkage. The consent field for users under 14 years of age is not applicable to this access channel because eligibility for Naver services is restricted to individuals aged 14 years or older.

### 2. Comparison of Usage Conditions by Access Channel

Following integration with Naver, Q-CODE offered three online access channels: the Q-CODE website, the Naver Pre-Arrival Quarantine Info Service, and the PHR app (Figure 2) [7]. The usage conditions for each channel are summarized in Table 1.

The Q-CODE website requires no login or authentication, has no age restrictions, and supports five languages: Korean, English, Chinese, Vietnamese, and Japanese. The Naver service requires membership registration and certificate issuance and is available only to individuals aged 14 years or older. However, it automatically populates personal information fields and supports two languages: Korean and English. Because the PHR app channel redirects users to the Q-CODE website, its input fields and supported languages are identical to those of the website.

Among the on-site declaration methods, kiosks are installed at selected international airports and support 16 languages: Korean, English, Simplified Chinese, Traditional Chinese, Japanese, Italian, French, German, Spanish, Russian, Arabic, Indonesian, Malay, Thai, Vietnamese, and Portuguese. Paper health questionnaires are available in three languages: Korean, English, and Simplified Chinese.

### 3. Usage by Quarantine Declaration Method

The distribution of the 148,861 quarantine declarations received in June–July 2026 by submission method is presented in Table 2. Of the total declarations, 2,741 (1.8%) were submitted on paper, while 146,120 (98.2%) were submitted electronically. Among all declarations, mobile (web) was the most frequently used method, accounting for 102,940 cases (69.2%), followed by arrivals hall kiosks with 36,627 cases (24.6%) and Naver with 6,553 cases (4.4%).

By month, Naver accounted for 4.1% (3,053 cases) of declarations in June and 4.8% (3,500 cases) in July. However, the two-month observation period was too short to support a robust analysis of usage trends. Furthermore, because the PHR app channel redirects users from the app’s main screen to the Q-CODE website, declarations submitted through this channel are included in the mobile (web) category in the issuance statistics. Consequently, its usage volume cannot be determined separately.

## Conclusion

To improve the usability of the Q-CODE, the KDCA launched the Naver Pre-Arrival Quarantine Info Service in December 2025 and added an access channel through the PHR app in June 2026. The Naver-linked service reduced the number of fields requiring manual entry from 11 to 5, and declarations submitted through Naver accounted for 4.4% of all quarantine declarations during June–July 2026.

The significance of the Q-CODE private-sector platform integration policy can be summarized in three points. First, rather than developing additional public-sector apps, the KDCA pursued a government-led approach to expanding access channels by linking existing private-sector platforms with public services, with the aim of improving user convenience. Second, the integration of private-sector platforms with the public quarantine information system incorporated considerations of consent for the collection and use of personal information and its provision to third parties, as well as data retention and destruction criteria. Third, while expanding access through private-sector platforms, the KDCA retained the existing Q-CODE website, which requires no separate login or authentication, and on-site declaration methods. During the analysis period, declarations submitted through arrivals hall kiosks accounted for 24.6% of all declarations, while paper-based declarations accounted for 1.8%. These findings suggest that existing declaration methods should be maintained alongside the expansion of access channels through private-sector platforms.

Furthermore, this policy has implications for preparedness and response to infectious disease emergencies. During the COVID-19 pandemic, the KDCA added a function to Q-CODE for registering post-entry test results, enabling it to manage the test results of 88,757 entrants from China and promptly provide the information to the relevant local governments [1]. Considering the experience of rapidly implementing additional functions in Q-CODE during the COVID-19 pandemic, establishing diverse access channels during non-emergency periods may provide a foundation for broader use of Q-CODE during future infectious disease emergencies. However, the effectiveness of this approach should be evaluated separately under actual emergency conditions.

This report has several limitations. First, the analysis of the Naver-linked service was based on only two months of data (June–July 2026), limiting the assessment of long-term usage trends; the potential influence of seasonal fluctuations in international travel demand also cannot be excluded. Second, because the utilization rate was calculated using the total number of quarantine declarations as the denominator, it does not reflect the actual adoption rate among individuals eligible to use the Naver service. Third, because the PHR app redirects users to the Q-CODE website, its usage was not separately recorded in operational statistics, precluding an assessment of the volume of use attributable to this access channel. Fourth, the analysis did not include direct measures of usability, such as data entry time, input error rate, completion rate, or user satisfaction. Finally, because the evaluation of private-platform integration was limited to a single platform, the findings may not be generalizable to other platforms.

Future efforts should include the continuous evaluation of service utilization, pre-entry data entry rates, and user experience among individuals eligible to use the service. Given Q-CODE’s public health objectives, ensuring that entrants submit the required quarantine information before arrival is particularly important. The pre-entry data entry rate should therefore be assessed using timestamps indicating when information was entered. Where necessary, guidance should be strengthened at pre-departure travel touchpoints, including airlines and travel industry channels. Furthermore, to prevent private-sector integration from creating dependence on a specific provider, the expansion of access to multiple platforms should proceed alongside the maintenance of universally accessible channels for non-members, foreign entrants, and digitally vulnerable groups. In particular, because registering for domestic private-sector platforms may be impractical for foreign entrants, multilingual guidance on the Q-CODE website should be strengthened, alongside guidance on completing paper health questionnaires [5].

## Figures and Tables

**Figure 1:**
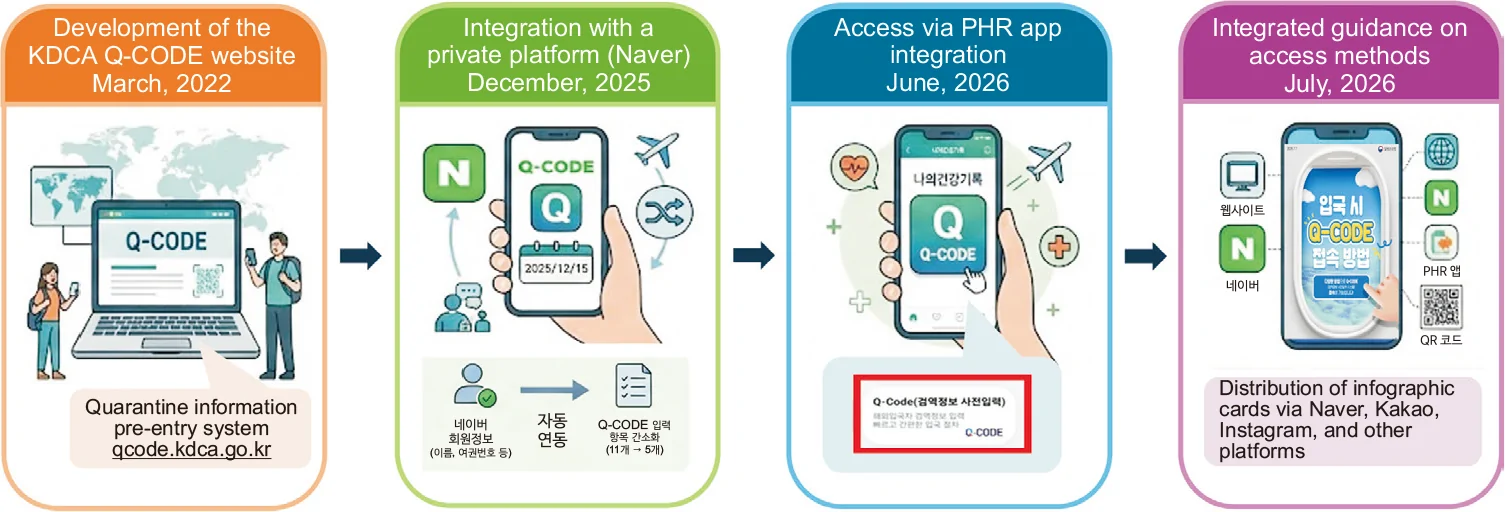
Progress in expanding online access channels for the Health Declaration System Upon Entry (Q-CODE) KDCA=Korea Disease Control and Pre­vention Agency; PHR=Personal Health Records. Data from Korea Disease Con­trol and Prevention Agency [7].

**Figure 2:**
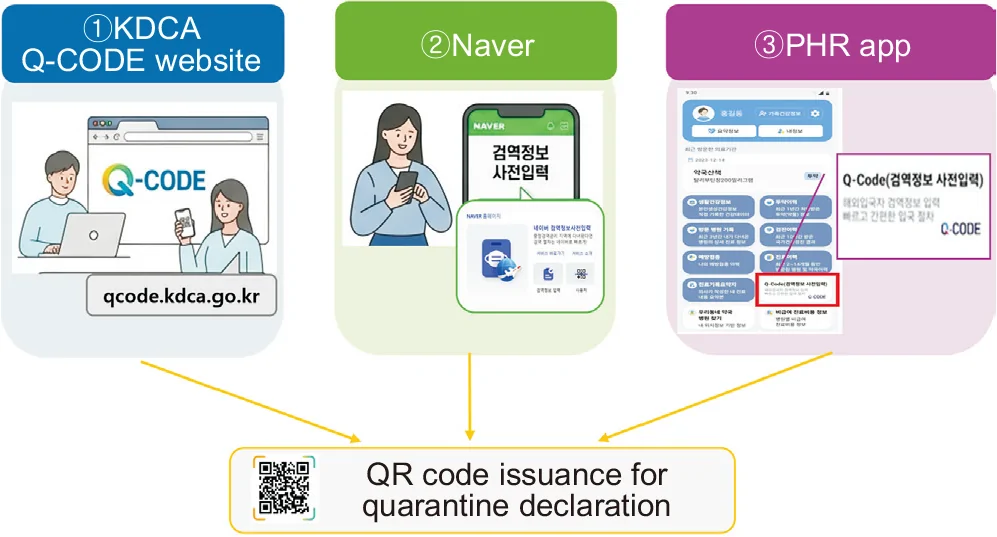
Health Declaration System Upon Entry (Q-CODE) online access channels KDCA=Korea Disease Control and Prevention Agency; PHR=Per­sonal Health Records. Data from Korea Disease Control and Pre­vention Agency [7].

**Table 1:** Comparison of access conditions and direct input items by online access route

Category		KDCA Q-CODE website	PHR app	Naver Pre-Arrival Quarantine Info Service
Supported platform		Responsive web (PC, mobile)	Mobile app	Mobile web/app
Restriction		None	Members of the PHR app	Naver members aged ≥14 yearsc)
Multilingual support		Five languages	Q-CODE webpage accessed through the PHR app	Two languages
Input information	Quarantine information	(1) Mode of transportation, (2) Country of final departure, (3) Date of arrival (scheduled) in the ROK, (4) Countries visited, (5) Symptoms (health status information)		(1) Mode of transportation, (2) Country of final departure, (3) Date of arrival(scheduled) in the ROK, (4) Countries visited, (5) Symptoms (health status information)
	Personal information	(6) Name in English, (7) Passport number, (8) Address of stay in the ROK, (9) Consent for individuals under 14 years of age, (10) Date of birth, (11) Contact information in the ROK		Automatically linked to Naver member information; no manual entry requireda)
	Totalb)	11 items		5 items

KDCA=Korea Disease Control and Prevention Agency; Q-CODE=Health Declaration System Upon Entry; PHR=Personal Health Records; ROK=Republic of Korea. ^a)^Items (6)–(8) require one-time entry only and do not need to be re-entered thereafter. ^b)^Total number of input items. ^c)^Naver members aged ≥14 years who have completed identity verification.

**Table 2:** Status of quarantine declaration methods used (June–July 2026)

Category	Overall	Mobile (web)a)	Kioskb)	Paper-basedc)	Naver
June 2026	75,344 (100.0)	51,715 (68.6)	19,042 (25.3)	1,534 (2.0)	3,053 (4.1)
July 2026	73,517 (100.0)	51,225 (69.7)	17,585 (23.9)	1,207 (1.6)	3,500 (4.8)
Total	148,861 (100.0)	102,940 (69.2)	36,627 (24.6)	2,741 (1.8)	6,553 (4.4)

Unit: case (%). Use of quarantine declaration methods among travelers subject to quarantine declaration arriving from designated quarantine management areas. ^a)^Declarations submitted through the KDCA Q-CODE website and the PHR app. ^b)^Kiosks located in selected international airports arrival areas. ^c)^Health questionnaire. KDCA=Korea Disease Control and Prevention Agency; Q-CODE=Health Declaration System Upon Entry; PHR=Personal Health Records.
