# Supplementary material for: Enhancing the Usability of the Health Declaration System Upon Entry (Q-CODE) through Private Platform Integration
Source: Public Health Wkly Rep. 2026 Nov 12;19(43):1824–37. doi: 10.56786/PHWR.2026.19.43.3 (PMC13759227; doi:10.56786/PHWR.2026.19.43.3)
Supplement: Translation

# Translation: 민간 플랫폼 연계를 통한 검역정보사전입력시스템(Q-CODE) 이용편의 개선 사례

**Authors:** 성주 유, 대일 박, 민경 채, 효준 주, 강현 이, 성순 김

**Affiliations:** 1 질병관리청 질병데이터과학분석관 역학데이터분석담당관; 2 질병관리청 감염병위기관리국 검역정책과

**Keywords:** 검역, 해외유입 감염병, 민관협력, 개인건강기록

## Abstract

**목적:**

질병관리청은 「검역법」에 따른 신고 대상 입국자가 체류‧경유지역과 건강상태 등 필요한 정보를 전자적으로 사전 입력할 수 있도록 2022년부터 검역정보사전입력시스템(Health Declaration System Upon Entry, Q-CODE)을 운영하고 있다. 질병관리청은 Q-CODE의 접근성과 이용 편의성을 높이기 위해 민간 플랫폼과의 연계를 검토해 왔으며, 2025년부터 본격적으로 추진하였다. 본 보고에서는 Q-CODE와 민간 플랫폼의 연계 및 접속경로 확대 과정을 기술하고, 연계 이후 초기 이용 현황과 향후 과제를 제시하고자 하였다.

**방법:**

2025년 12월 15일 민간 플랫폼인 네이버와 Q-CODE를 연계하여 개인정보 항목을 네이버 회원 정보와 연동하였고, 2026년 6월에는 나의건강기록(Personal Health Records, PHR) 앱을 통해 Q-CODE에 접근할 수 있도록 접속경로를 추가하였다. 연계 이후 초기 이용 현황을 파악하기 위해 2026년 6월 1일부터 7월 31일까지 접수된 검역 신고 148,861건을 신고 수단별로 분석하였다.

**결과:**

네이버 연계 서비스에서 이용자가 직접 입력하는 항목은 기존 11개에서 5개로 감소하였다. 전체 검역 신고 148,861건 중 네이버를 통한 신고는 6,553건(4.4%)이었으며, 월별로는 6월 4.1% (3,053건), 7월 4.8% (3,500건)였다.

**결론:**

Q-CODE와 민간 플랫폼(네이버)과의 연계를 통해 이용자가 직접 입력하는 항목을 줄일 수 있었으며, Q-CODE의 온라인 접속경로를 기존 질병관리청 누리집에서 네이버 및 PHR 앱으로 확대하였다. 기존 Q-CODE 누리집도 별도의 로그인이나 인증 없이 이용할 수 있도록 유지하여 사용자의 이용 혼선을 예방하였다. 향후 다양한 민간 플랫폼으로 연계를 확대하되, 특정 플랫폼에 종속되지 않는 선택적‧보편적 접근성을 함께 보장할 필요가 있다.

**핵심요약**

**① 이전에 알려진 내용은?**

질병관리청은 해외유입 감염병 검역을 위해 입국자가 건강상태 등 필요한 정보를 입국 전에 전자적으로 제출할 수 있는 검역정보사전입력시스템(Health Declaration System Upon Entry, Q-CODE)을 운영하고 있다.

**② 새로이 알게 된 내용은?**

민간 플랫폼인 네이버와 Q-CODE 연계를 통해 이용자가 직접 입력해야 하는 항목이 11개에서 5개로 감소하였다. 2026년 6–7월 전체 검역 신고 148,861건 중 네이버를 통한 신고는 6,553건(4.4%)이었다.

**③ 시사점은?**

국민이 이용하는 민간 플랫폼과 공공 검역 서비스의 연계는 이용 가능한 접속경로와 편의성을 개선하는 방안이 될 수 있다.

## 서 론

해외여행의 증가와 국가 간 이동의 일상화에 따라 해외에서 유입되는 감염병을 조기에 확인하고 대응하기 위한 검역의 중요성이 커지고 있다. 질병관리청은 「검역법」에 따른 신고 대상 입국자로부터 체류‧경유지역과 건강상태 등 검역에 필요한 정보를 제출받고 있으며, 해당 정보는 입국 단계의 검역조사와 이후 접촉자 추적조사 등 필요한 공중보건조치에 활용된다[1].

코로나바이러스감염증-19(코로나19) 대응 초기에는 코로나19의 국내 유입 및 지역사회 전파를 조기 차단하기 위해 한국에 입국하는 모든 입국자에 대해 발열 체크, 건강상태질문서‧특별검역신고서 제출, 사후모니터링 등을 시행하였고, 국내‧외 코로나19 확산에 따라 입국 전 음성확인서 제출, 예방접종력에 따라 자가 또는 임시생활시설 격리 등의 조치를 시행하였다. 이후 코로나19 대응을 위해 전 세계 모든 국가를 검역관리지역으로 지정하게 되면서 모든 입국자에 대해 종이 형태의 건강상태질문서를 징구하게 되었다. 이로 인해 검역 대상자가 급증하게 되면서 모든 입국자로부터 종이 서류를 징구하고, 검역조사를 위한 상당히 많은 시간이 소요되어 입국자 불편, 검역관의 업무 부담 등이 발생하였다.

이에 질병관리청은 자가격리 앱을 활용한 특별입국 절차 방식을 중단한 후(2022년 2월 21일), 검역을 통한 해외유입 차단 효과 및 입국객의 입국 대기를 최소화하기 위해 2022년 3월 21일 인천공항을 시작으로 검역정보사전입력시스템(Health Declaration System Upon Entry, Q-CODE)을 도입하여 입국자가 건강상태 등 검역에 필요한 정보를 입국 전에 전자적으로 입력하고 QR 코드를 발급받을 수 있도록 하였다. 입국장 내 설치된 키오스크와 Q-CODE를 이용할 수 있게 되면서 평균 검역 소요시간이 2분–4분 30초에서 45초 수준으로 단축되었으며, 2024년 1월 23일 「검역법」 제12조 개정을 통해 검역정보시스템을 통한 서류 제출의 법적 근거도 명확히 하였다[1].

2023년 5월 세계보건기구 국제공중보건위기상황 해제, 6월 위기경보 경계 하향, 코로나19의 제4급감염병 전환 등 국내외 동향을 고려하여 코로나19 위기경보 수준이 경계로 하향 조정되면서 전 세계로 지정되었던 코로나19 검역관리지역 및 그에 따른 검역 조치가 해제되었다. 코로나19의 단계적 일상 회복에 따라 2023년 11월 질병관리청에서 진행한 국민 인식조사에서 Q-CODE 이용 경험자 505명 중 76.8%가 이용에 만족한다고 응답하였으며, 시스템 자체에 대한 이용자 평가는 긍정적으로 나타났다. 이러한 결과는 Q-CODE 자체에 대한 이용자 수용성이 일정 수준 확보되었음을 보여주고 있다. 반면, 질병관리청 Q-CODE 누리집은 QR 코드 발급을 위해 입력해야 하는 항목이 너무 많고, 인터페이스가 불편하다는 이용자의 의견이 제기되어 왔으며, 질병관리청은 입력 항목 간소화, 불필요한 안내 삭제, 중복 입력 항목 제외 등을 통해 이용자 편의성 개선을 위해 노력하였다[1].

국외에서도 일본의 Visit Japan Web과 싱가포르의 SG Arrival Card 등 입국자가 필요한 정보를 입국 전에 디지털 방식으로 제출할 수 있도록 하는 서비스가 운영되고 있다[2,3]. 이러한 국내외 여건을 고려할 때, 디지털 전환 환경에서는 Q-CODE 자체의 기능 개선과 함께 이용자가 검역 신고 서비스에 보다 쉽게 접근할 수 있도록 접속경로를 다양화하는 방안을 검토할 필요가 있다. 이에 본 보고에서는 2025년부터 추진한 Q-CODE와 민간 플랫폼의 연계 및 접속경로 확대 과정과 운영 구조를 기술하고, 연계 이후의 초기 이용 현황을 분석하여 향후 개선 과제를 제시하고자 하였다.

## 방 법

### 1. 민간 플랫폼 연계 추진

질병관리청은 이용자 편의성 개선을 위해 민간 플랫폼과의 연계를 추가 검토하였다. 당시 특정 플랫폼으로 국한하지 않고 다양한 민간 플랫폼과의 협력을 타진하여 순차적으로 서비스를 개시하는 방식을 적용하였다.

첫 사례로 2025년 12월 15일 네이버 출입증과 Q-CODE를 연계한 ‘네이버 검역정보사전입력 서비스’를 개시하였다[4]. 네이버 사례의 핵심은 반복 입력되던 개인정보 항목을 본인인증이 완료된 네이버 회원 정보와 연동하여 기본정보는 자동 입력하고, 검역정보만 직접 입력하여 검역 신고를 완료할 수 있도록 업무 절차를 설계하여 편의성을 향상시키고자 하였다.

또한, 설계 단계에서 개인정보 보호 및 안전성 확보를 주요한 조건으로 설정하였다. Q-CODE는 해외 여행객에게 개인정보(이름, 여권번호) 및 민감정보(방문국가, 건강상태 등)를 요구하는데, 네이버 검역정보사전입력 서비스 이용 전 개인정보 수집‧이용 및 제3자 제공 동의 절차를 적용하고, 서버에 저장된 정보의 보유‧파기 기준을 별도로 설정하였다. 또한 네이버에 저장된 검역정보사전입력 데이터는 한국 도착 예정일 다음날 자정에 자동으로 파기되도록 하였다.

### 2. 접속경로 확대

온라인 접속경로는 ① Q-CODE 누리집 직접 접속, ② 네이버 검역정보사전입력 서비스, ③ 나의건강기록(Personal Health Records, PHR) 앱 3가지이며, 2026년 6월에 추가한 PHR 앱 경로는 앱 메인화면에서 Q-CODE 누리집으로 연결되도록 구성하였다. 접속경로 ①은 별도의 로그인이나 인증 없이 이용할 수 있도록 유지하였다. 이는 민간 플랫폼 연계가 기존 이용자의 편의를 돕는 선택적 부가 기능이며, 특정 플랫폼 가입 여부가 검역 신고의 전제 조건이 되지 않도록 하기 위함이다. 이에 따라 민간 플랫폼 미가입자, 만 14세 미만 이용자, 외국인 입국자도 제약 없이 검역 신고를 할 수 있도록 하였다.

온라인 접속 외에 현장에서도 검역 신고를 할 수 있도록 입국 전 기내에서 서면 건강상태질문서를 배부하고, 입국장 검역대에 키오스크를 설치‧운영하고 있다. 특히 스마트기기 사용이 익숙하지 않은 입국자에게는 서면 건강상태질문서를 함께 안내하여, 디지털 접근성 수준에 관계없이 검역 신고가 가능하도록 하였다[5].

한편, 지원 언어는 경로별 이용 대상을 고려하여 구성하였다. Q-CODE 누리집과 입국장 키오스크는 다양한 국적의 입국자가 이용하는 공공 검역수단이므로 여러 언어를 지원하고, 네이버 서비스는 이용 대상의 특성을 고려하여 한국어와 영어로 제공하였다.

### 3. 대국민 홍보

신규 접속경로의 이용을 안내하기 위해 서비스 개시와 함께 대국민 홍보를 시행하였다. 2025년 12월 네이버 연계 서비스 개시에 맞추어 질병관리청은 보도자료를 배포하고, 민간 플랫폼 사업자와 공동으로 홍보자료를 제작하였다[4]. 2026년 7월에는 Q-CODE의 세 가지 온라인 접속경로 이용 방법을 안내하는 카드뉴스를 제작‧배포하였으며, PHR 앱을 통한 안내도 진행하였다(그림 1) [6,7].

## 결 과

### 1. 민간 플랫폼 연계를 통한 입력 항목 축소

정책 추진 결과, 네이버 검역정보사전입력 서비스에서는 회원 정보 연계를 통해 이용자가 직접 입력해야 하는 항목이 기존 11개에서 5개로 감소하였다(표 1). 직접 입력하지 않아도 되는 항목은 영문성명, 여권번호, 체류주소, 생년월일, 국내연락처 및 만 14세 미만 동의 항목이다. 이 중 영문성명‧여권번호‧체류주소는 기존 Q-CODE 누리집에서도 최초 입력 후 재입력이 필요하지 않았으나, 네이버 연계 서비스에서는 최초 입력 과정도 생략된다. 생년월일과 국내연락처는 회원 정보 연계를 통해 직접 입력을 대체하였으며, 만 14세 미만 동의 항목은 네이버 서비스 이용 대상이 만 14세 이상으로 제한됨에 따라 해당 경로에서는 적용되지 않는다.

### 2. 접속경로별 이용 조건 비교

네이버 연계 이후 Q-CODE 온라인 접속경로는 Q-CODE 누리집, 네이버 검역정보사전입력 서비스, PHR 앱의 3종으로 확대되었으며(그림 2) [7], 경로별 이용 조건은 표 1과 같다.

Q-CODE 누리집은 로그인이나 인증 절차가 없고 연령 제한 없이 이용할 수 있으며, 5개 국어(한국어, 영어, 중국어, 베트남어, 일본어)를 지원한다. 네이버는 회원 가입과 인증서 발급이 필요하고 만 14세 이상만 이용할 수 있으나, 개인정보 항목이 자동으로 입력되며 한국어와 영어 2개 국어를 제공한다. PHR 앱 경로는 Q-CODE 누리집으로 연결되므로 입력 항목과 지원 언어가 누리집과 같다.

현장 신고 수단 중 입국장 키오스크는 일부 국제공항에 설치되어 있으며 16개 국어(한국어, 영어, 중국어[간체], 중국어[번체], 일본어, 이탈리아어, 프랑스어, 독일어, 스페인어, 러시아어, 아랍어, 인도네시아어, 말레이어, 태국어, 베트남어, 포르투갈어)를 지원하고, 서면 건강상태질문서는 3개 국어(한국어, 영어, 중국어[간체])로 제공된다.

### 3. 검역 신고 수단별 이용 현황

2026년 6–7월 접수된 검역 신고 148,861건의 신고 수단별 분포는 표 2와 같다. 전체 신고 중 서면 방식은 2,741건(1.8%)으로, 146,120건(98.2%)이 전자적 방식으로 이루어졌다. 검역 신고 수단별 이용 현황은 모바일(웹)이 102,940건(69.2%)으로 가장 많았고, 입국장 키오스크 36,627건(24.6%), 네이버 6,553건(4.4%) 순이었다.

월별 네이버 경로 이용 비율은 6월 4.1% (3,053건), 7월 4.8% (3,500건)이다. 다만 관찰 기간이 2개월로 짧아 별도 추세 분석을 하기엔 제한이 있다. 또한, PHR 앱 경로는 앱 메인화면에서 누리집으로 연결되는 구조이므로 발급 통계상 모바일(웹)에 포함되어, 해당 경로의 이용 규모는 별도로 확인되지 않는다.

## 결 론

질병관리청은 Q-CODE 이용편의 개선을 위해 2025년 12월 네이버와 연계한 검역정보사전입력 서비스를 개시하고, 2026년 6월 PHR 앱에서 Q-CODE로 접속할 수 있는 경로를 추가하였다. 네이버 연계 서비스에서 이용자가 직접 입력하는 항목은 기존 11개에서 5개로 감소하였으며, 네이버를 통한 신고는 전체 검역 신고의 4.4%였다.

이번 Q-CODE 민간 플랫폼 연계 정책의 의의는 세 가지로 정리할 수 있다. 첫째, 공공 앱을 추가 구축하기보다 정부 주도로 기존 민간 플랫폼과 공공 서비스를 연계하여 새로운 접근경로를 마련하고, 그 안에서 국민 이용 편의성을 높일 수 있는 방안도 고려하였다는 점이다. 둘째, 민간 플랫폼과 공공 검역정보시스템을 연계하는 과정에서 개인정보 수집‧이용과 제3자 제공에 대한 동의 절차 및 정보의 보유‧파기 기준을 함께 고려하였다는 점이다. 셋째, 민간 플랫폼을 통한 접속경로를 추가하면서도 별도의 로그인이나 인증 없이 이용할 수 있는 기존 Q-CODE 누리집과 현장 신고 수단을 함께 유지하였다는 점이다. 분석 기간 중 입국장 키오스크를 통한 신고가 24.6%, 서면 신고가 1.8%를 차지한 점을 고려할 때, 민간 플랫폼 경로의 확대와 함께 기존 신고 수단을 유지할 필요성을 시사한다.

또한 이번 정책은 감염병 위기 대응의 관점에서도 함의를 갖는다. 코로나19 유행 시기 질병관리청은 Q-CODE에 입국 후 검사결과 등록 기능을 추가하여 중국발 입국자 88,757명의 검사결과를 관리하고 관할 지방자치단체에 신속히 정보를 제공한 바 있다[1]. 코로나19 유행 당시 Q-CODE에 추가 기능을 신속하게 적용한 경험을 고려할 때, 평시에 다양한 접속경로를 확보하는 것은 향후 감염병 위기 시 Q-CODE 활용 범위를 확대하기 위한 기반으로 활용될 가능성이 있다. 다만 이러한 효과는 실제 위기 상황에서 별도로 평가할 필요가 있다.

본 보고에는 몇 가지 한계가 있다. 첫째, 네이버 연계 서비스의 이용 현황을 2026년 6–7월의 2개월 자료로 분석하여 장기적인 이용 추이를 판단하기 어렵고 계절별 국제여행 수요 변동의 영향을 배제할 수 없다. 둘째, 전체 검역 신고를 분모로 이용 비율을 산출하였으므로 네이버 서비스 이용이 가능한 대상자에서의 실제 서비스 채택률을 의미하지 않는다. 셋째, PHR 앱은 Q-CODE 누리집으로 연결되는 구조로 운영 통계상 별도 집계되지 않아 해당 경로의 이용 규모를 평가할 수 없었다. 넷째, 입력 소요시간, 입력 오류율, 완료율 및 이용자 만족도 등 이용 편의성을 직접 평가할 수 있는 지표가 포함되지 않았다. 마지막으로 현재 민간 플랫폼 연계 사례가 한 곳에 한정되어 있어 일반화에 한계가 있다.

향후에는 서비스 이용 가능 대상자에서의 이용률과 사전 입력률 및 이용자 경험을 지속적으로 평가할 필요가 있다. Q-CODE의 정책 목적을 고려하면 입국 전에 필요한 검역정보를 입력하도록 안내하는 것이 중요하다. 실제 입력 시점 자료를 활용하여 도착 전 사전 입력 비율을 확인하고, 필요할 경우 항공사‧여행업계 등 출국 전 여행 접점에서의 안내를 강화할 필요가 있다. 또한 민간 플랫폼 연계가 특정 사업자에 대한 종속으로 인식되지 않도록 복수 플랫폼으로의 확대와 비회원‧외국인‧디지털 취약계층을 위한 보편적 경로 유지가 병행되어야 한다. 특히 외국인 입국자의 경우 국내 민간 플랫폼 회원 가입이 현실적으로 어려우므로, 누리집 경로의 다국어 안내와 서면 건강상태질문서 안내를 함께 강화할 필요가 있다[5].

## Figures and Tables

**그림 1:** 검역정보사전입력시스템(Q-CODE) 온라인 접속경로 확대 추진 경과 PHR=나의건강기록. Data from Korea Disease Control and Prevention Agency [7].

**그림 2:** 검역정보사전입력시스템(Q-CODE) 온라인 접속경로 Data from Korea Disease Control and Prevention Agency [7].

**표 1:** 온라인 접속경로별 이용 조건 및 직접 입력 항목 비교

구분		질병관리청 Q-CODE 누리집	나의건강기록 앱	네이버 검역정보사전입력
지원 플랫폼		반응형 웹(PC, 모바일)	모바일 앱	모바일 웹/앱
제한조건		제한 없음	나의건강기록 앱 회원	만 14세 이상 네이버 회원c)
다국어 지원		5종	나의건강기록 앱에서 Q-CODE 누리집 페이지를 호출하는 방식	2종
입력 정보	검역 정보	①탑승수단, ②최종출발국가, ③한국 도착(예정)일자, ④방문국가, ⑤증상(건강상태정보)		①탑승수단, ②최종출발국가, ③한국 도착(예정)일자, ④방문국가, ⑤증상(건강상태정보)
	개인 정보	⑥영문성명, ⑦여권번호, ⑧체류주소, ⑨만 14세 미만 동의, ⑩생년월일, ⑪국내연락처		네이버 회원 정보와 자동 연계되어 입력 불필요a)
	합계b)	11항목		5항목

Q-CODE=검역정보사전입력시스템. ^a)^⑥–⑧ 항목은 최초 1회 입력 후 재입력 불필요. ^b)^입력정보 합계. ^c)^만 14세 이상 본인인증을 완료한 네이버 회원.

**표 2:** 2026년 6–7월 검역 신고 수단별 이용 현황

구분	전체	모바일(웹)a)	키오스크b)	서면c)	네이버
2026년 6월	75,344 (100.0)	51,715 (68.6)	19,042 (25.3)	1,534 (2.0)	3,053 (4.1)
2026년 7월	73,517 (100.0)	51,225 (69.7)	17,585 (23.9)	1,207 (1.6)	3,500 (4.8)
합계	148,861 (100.0)	102,940 (69.2)	36,627 (24.6)	2,741 (1.8)	6,553 (4.4)

단위: 건(%). 중점검역관리지역에서 입국한 검역 신고 대상자의 검역 신고 수단 이용 현황. ^a)^질병관리청 검역정보사전입력시스템(Q-CODE) 누리집 및 나의건강기록 앱을 이용하여 검역 신고한 현황. ^b)^일부 국제공항 내 설치된 키오스크. ^c)^건강상태질문서.
